# Transcriptional repression of p21 by EIF1AX promotes the proliferation of breast cancer cells

**DOI:** 10.1111/cpr.12903

**Published:** 2020-09-14

**Authors:** Yuhuan Li, Lu Guo, Sunyang Ying, Gui‐Hai Feng, Ying Zhang

**Affiliations:** ^1^ State Key Laboratory of Stem Cell and Reproductive Biology Institute of Zoology Chinese Academy of Sciences Beijing China; ^2^ University of Chinese Academy of Sciences Beijing China; ^3^ Institute for Stem Cell and Regeneration Chinese Academy of Sciences Beijing China

## Abstract

**Objective:**

Dysregulation of the cell cycle is associated with the progression of malignant cancer, but its precise functional contribution is unknown.

**Materials and Methods:**

The expression of EIF1AX in breast cancer tissues was detected by qRT‐PCR and immunohistochemistry staining. Colony formation and tumour xenograft assays were used to examine the tumorigenesis‐associated function of EIF1AX in vitro and in vivo. RNA‐Seq analysis was used to select the downstream target genes of EIF1AX. Flow cytometry, ChIP and luciferase assays were used to investigate the molecular mechanisms by which EIF1AX regulates p21 in breast cancer cells.

**Results:**

EIF1AX promoted breast cancer cell proliferation by promoting the G1/S cell cycle transition. A mechanistic investigation showed that EIF1AX inhibited the expression of p21, which is an essential cell cycle regulator. We identified that the transcriptional regulation of p21 by EIF1AX was p53‐independent. Clinically, EIF1AX levels were significantly elevated in breast cancer tissues, and the high level of EIF1AX was associated with lower survival rates in breast cancer patients.

**Conclusions:**

Our results imply that EIF1AX may play a key role in the incidence and promotion of breast cancer and may, thus, serve as a valuable target for breast cancer therapy.

## Graphical abstract

Dysregulation of cell cycle has been implicated in the progression of malignant cancer, but the precise functional contributions are uncertain. In this issue, Li *et al* show that EIF1AX inhibits the transcription of critical cell‐cycle regulator p21 in a p53‐independent way and then promotes breast cancer cell proliferation and tumorigenesis through promoting the G1/S transition in cell cycle.

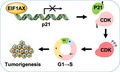

## 1 INTRODUCTION

Dysregulation of cell cycle progression is common in tumorigenesis; it leads to the over‐proliferation of cells. Cyclin‐dependent kinases (CDKs) regulate the cell cycle, and their activity is partially inhibited by CDK inhibitors, including p21 (also known as CDKN1A). p21 has been identified as a significant cell cycle regulator at the G1/S and G2/M transition.^1^, ^2^ Increased p21 expression inhibits cell growth and proliferation.^3^, ^4^

Expression of the p21 gene is induced in cells in response to various stresses in a p53‐dependent or p53‐independent manner. In the face of DNA damage and many other cellular stressors, p21 levels are elevated in a p53‐dependent manner, contributing to cell proliferation arrest. In addition to p53, an array of tumour suppressor proteins and oncogenes can induce p21 expression by binding to specific sites on the p21 promoter.^5^, ^6^ In any case, p21 causes cell cycle arrest and inhibits CDK activity, which both are essential for tumour suppressor gene, Rb inactivation. However, the molecular mechanisms underlying the functions of p21 remain unclear.

EIF1AX is encoded on human chromosome X^7^, ^8^; it is essential for assembling the 43S pre‐initiation complex (PIC).^9^ EIF1AX mutations have been reported in many cancers^7^, ^10^, ^11^, ^12^, ^13^ and these mutations are presumed to result in increased or altered function, owing to their preference for specific substitutions in the C‐ and N‐terminal tails. EIF1AX regulates cell proliferation in bovine mammary epithelial cells.^14^ Despite its mutations in many cancers and its role in promoting mammary epithelial cell proliferation, the functions of EIF1AX in cancer, and the cellular mechanisms underlying these functions are poorly understood.

Here, we report that EIF1AX promotes breast cancer cell proliferation by promoting the G1/S phase transition through the transcriptional repression of p21 in a p53‐independent manner and, consequently, has a marked effect on the incidence and progression of breast cancer.

## 2 MATERIALS AND METHODS

### 2.1 Cell culture

Human breast cancer cell lines and HCT‐116 cell line were cultured in Modified Eagle's medium (DMEM) + 10% fetal bovine serum (FBS).

### 2.2 Tissue specimens and immunohistochemistry (IHC) staining

This study was approved by the Ethics Committee of the Institute of Zoology, Chinese Academy of Sciences, and all patients provided informed consent before surgery. Beijing 301 Military General Hospital (Beijing, China) offered human breast carcinoma tissues. IHC analysis was performed as described previously,^15^ using anti‐EIF1AX antibodies (11649‐2‐AP; Proteintech).

### 2.3 Gene expression assay

TRIzol Reagent (15596‐018; Invitrogen) was used for total RNA extraction, and then total RNA was reverse‐transcribed into cDNA with reverse transcription system (A3500; Promega). Quantitative real‐time reverse transcription polymerase chain reaction (qRT‐PCR) was performed using SYBR Premix Ex Taq kit (RR420A; TaKaRa) on a Stratagene Mx3000P quantitative PCR system (Genetimes Technology). The PCR program was 95°C, 10 minutes; 95°C, 15 seconds, 60°C, 1 minute, 40 cycles. There were three technical repetitions for all the reactions. The primers used in the study are listed in Table S1.

### 2.4 Western blotting

Western blotting was performed as described previously using the following antibodies: anti‐α‐tubulin (T6199; Sigma), anti‐EIF1AX (ab177939; Abcam), anti‐p21 (K0081‐3; MBL), anti‐p53 (sc‐47698; Santa Cruz Biotechnology), anti‐cyclin D1 (2978S; Cell Signaling Technology) and anti‐P‐Rb (8180S; Cell Signaling Technology).

### 2.5 Oligonucleotide transfections

RiboBio Co. synthesized and purified all commercial EIF1AX targeting small interfering RNAs (siRNAs). Transfections of the siRNAs were performed using Lipofectamine 3000 (Invitrogen), following the manufacturer's instructions. The siRNA sequences used are listed in Table S1.

### 2.6 Flow cytometry

The cultured cells were digested with trypsin, washed with cold phosphate‐buffered saline (PBS) and finally fixed with cold 70% ethanol in PBS. Cells were pelleted and resuspended in cold PBS before staining. 2 μg/mL Bovine pancreatic RNase (Sigma‐Aldrich) was added and incubated at 37°C for 30 minutes. Next, the cells were treated with 20 μg/mL propidium iodide (Sigma‐Aldrich) for 20 minutes. The cell cycle profile of each cell sample (2 × 10^4^ cells) was analysed by A FACSCalibur flow cytometer (BD Biosciences).

### 2.7 Colony formation assay

Breast cancer cells MCF‐7 were infected with lentiviral constructs encoding EIF1AX or EIF1AX shRNA. After 14 days, colonies were stained with 0.1% crystal violet solution, and the images were obtained using a light microscope.

### 2.8 Cell proliferation assay

MCF‐7 cells were digested with trypsin and plated into 96‐well plates at a density of 1 × 10^4^ cells/well. Cell Counting Kit‐8 (CCK8, Promega) was used to perform cell proliferation assay.

### 2.9 Tumour xenograft assay

MCF‐7 cells overexpressing EIF1AX or expressing EIF1AX‐targeted shRNA (5 × 10^6^ cells in 200 μL PBS) were injected into the mammary fat pads of 8‐week‐old athymic BALB/c mice under aseptic conditions. The tumours were weighted at week 8 after the injection.

### 2.10 Chromatin immunoprecipitation (ChIP) assay

Soluble chromatin from MCF‐7 cells and p53^+/+^ or p53^−/−^ HCT‐116 cells was immunoprecipitated with anti‐EIF1AX or control rabbit normal IgG antibodies. The final extracted DNA was amplified by qRT‐PCR using specific primers that cover the proximal promoter region of p21 gene. The primers used in the study are listed in Table S1.

### 2.11 Accession numbers

The raw data for RNA‐Seq have been deposited in the Genome Sequence Archive of the Beijing Institute of Genomics, Chinese Academy of Sciences under project CRA002538, which is publicly accessible at http://gsa.big.ac.cn/.

### 2.12 Statistical analyses

Data shown are presented as the mean ± SD of three or more independent experiments. The differences between two groups were determined by Student's *t* test. Scatter plots and Pearson's correlation analysis were used for statistical analysis. The cor.test function of R was used to calculate the correlation coefficients and the corresponding *P*‐values.

## 3 RESULTS

### 3.1 EIF1AX is elevated in breast cancer cell lines and human mammary tumours

**Figure 1:**
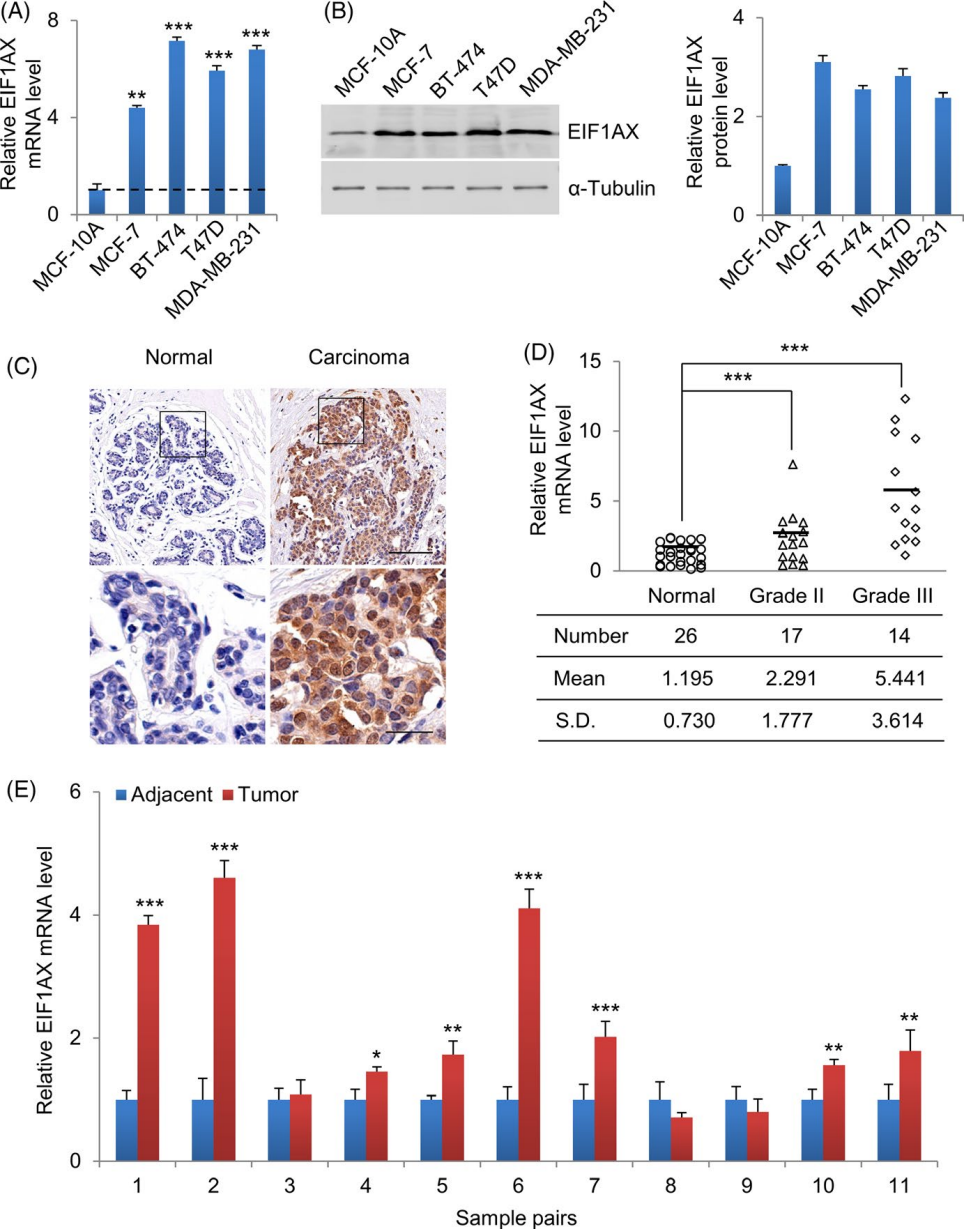
EIF1AX expression is elevated in human breast cancer cell lines and breast tumours. A, The expression of EIF1AX in different human breast cancer cell lines using qRT‐PCR, MCF‐10A served as control. B, The expression of EIF1AX in different human breast cancer cell lines using Western blotting, MCF‐10A served as control, α‐Tubulin served as the internal control. C, Representative IHC staining of EIF1AX protein in human breast carcinoma tissue (right panel) and adjacent normal breast tissue (left panel). The carcinoma tissue showed brown EIF1AX staining. The staining was nearly absent in normal breast tissue. Scale bar, 100 μm (upper panel), 25 μm (lower panel). D, Expression of EIF1AX in adjacent normal and breast carcinoma tissue samples. Relative mRNA expression of EIF1AX was measured using qRT‐PCR with GAPDH as the internal control. E, qRT‐PCR analysis of the expression of EIF1AX levels in breast carcinoma and adjacent normal tissues. Data are presented as mean ± SD, Student's *t* test, *P < .05, **P < .01, ***P < .001

Firstly, the mRNA expression and protein expression of EIF1AX were examined in normal human breast epithelial cells (MCF‐10A) and several breast cancer cell lines. As shown in Figure 1A,B, EIF1AX was higher in breast cancer cells than that in normal breast epithelial cells at both mRNA and protein levels (Figure 1A,B). Using immunohistochemistry, we detected the expression of EIF1AX in paired adjacent normal mammary tissues and mammary carcinoma tissues. Compared with normal mammary epithelial cells, the breast cancer cells displayed stronger EIF1AX staining in both the nucleus and cytoplasm (Figure 1C).

To illustrate the relationship between the high expression level of EIF1AX and the incidence and progression of breast tumours, mRNA expression of EIF1AX in primary tumours was detected using qRT‐PCR. Thirty‐one carcinoma tissues and twenty‐six adjacent normal tissues from breast cancer patients were collected. We found that EIF1AX mRNA was significantly upregulated in carcinoma tissues and the expression of EIF1AX was positively correlated with histological grades (Figure 1D). Compared with adjacent normal tissues, EIF1AX mRNA had increased in the majority of breast carcinoma tissues (Figure 1E).

### 3.2 EIF1AX promotes breast cancer cell proliferation and tumorigenesis

**Figure 2:**
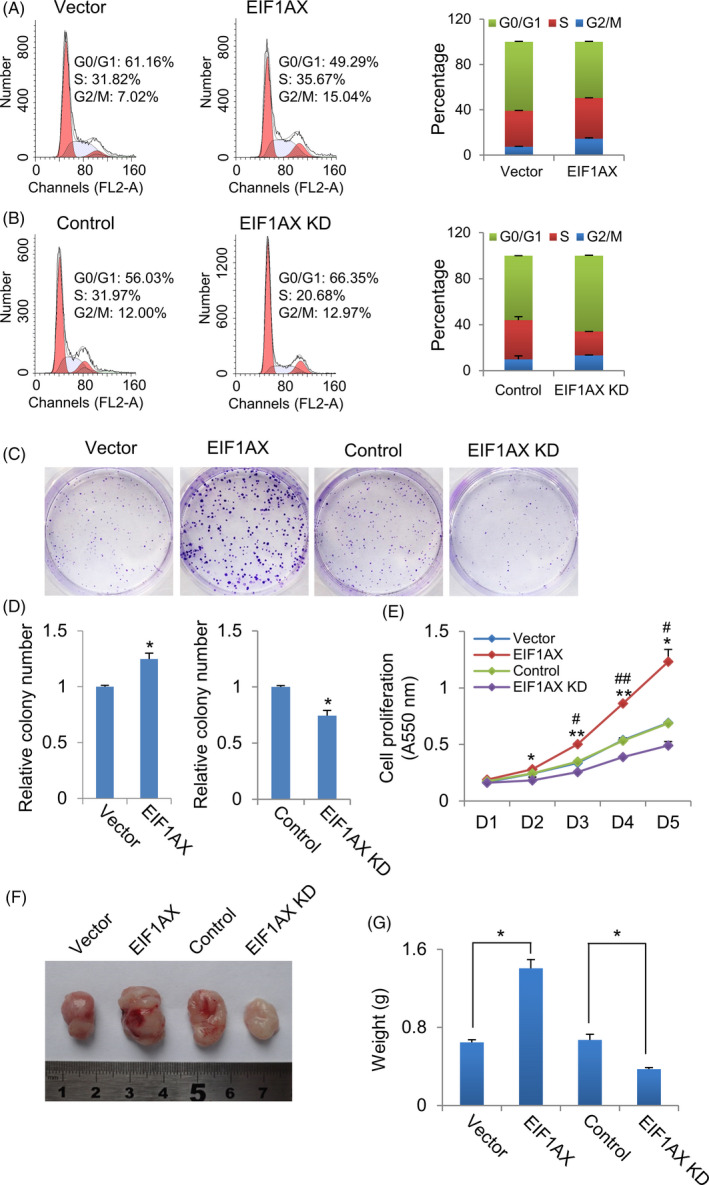
EIF1AX promotes breast cancer cell proliferation and tumorigenesis. A, The effect of EIF1AX overexpression on cell cycle progression. Cell cycle analysis of control and EIF1AX overexpressed MCF‐7 cells by flow cytometry. The right panel data are presented as the mean ± SD from three independent experiments. The left panel shows representative results. B, The effect of EIF1AX knock‐down on cell cycle progression. Cell cycle analysis of control and EIF1AX knock‐down MCF‐7 cells using flow cytometry. The right panel data are presented as the mean ± SD from three independent experiments. Representative results are shown in the left panel. C and D, Colony formation assay. Represent images of EIF1AX overexpressed and knock‐down MCF‐7 cell colonies (C). Statistics of the colony number of each group (D). Data are presented as the mean ± SD from triplicate experiments. *P < .05, Student's *t* test. E, The effect of EIF1AX on cell proliferation. Cell proliferation analysis of EIF1AX overexpressed and knock‐down MCF‐7 cells. Data are expressed as the mean ± SD from triplicate experiments. *P < .05; **P < .01, compared with empty vector. ^#^ *P* < .05; ^##^ *P* < .01, compared with control siRNA, Student's *t* test. F and G, EIF1AX promotes breast tumorigenesis. Athymic mice were injected with EIF1AX‐overexpressing or EIF1AX shRNA‐expressing MCF‐7 cells. Representative tumour images (F) and the average tumour mass (G). Each point represents the mean ± SD *P < .05, Student's *t* test

We hypothesized that the abnormal expression of EIF1AX in breast cancer cell lines and mammary tumours may have pathological relevance. To test this hypothesis, we investigated whether EIF1AX influenced breast cancer cell proliferation and breast tumorigenesis. First, we detected the regulation of the cell cycle in breast cancer cells with EIF1AX overexpression or knock‐down. Flow cytometry analysis revealed that the G0/G1 phase to the S + G2/M phase transition of cells increased when EIF1AX was overexpressed (Figure 2A). Consistent with this result, an accumulation of G0/G1 phase cells was found in EIF1AX knock‐down cells (Figure 2B). In brief, these results indicate that EIF1AX facilitates the G1/S transition, resulting in the promotion of breast cancer cell proliferation.

To illustrate the role of EIF1AX in breast cancer tumorigenesis in vitro, we performed colony formation assays and found that the number of colonies formed increased following EIF1AX overexpression, whereas EIF1AX knock‐down reduced the number of colonies formed (Figure 2C,D). To further confirm the effect of EIF1AX on cell proliferation, we performed cell proliferation assays using the CCK8 assay. Consistent with the above results, we found that EIF1AX overexpression increased the growth rate, while EIF1AX knock‐down decreased the growth rate of breast cancer cells (Figure 2E).

To confirm the role of EIF1AX in breast cancer tumorigenesis in vivo, EIF1AX overexpression or EIF1AX knock‐down MCF‐7 cell lines and their control cells were implanted into the mammary fat pads of athymic BALB/c mice. We measured the growth of the implanted tumours (n = 6) during an 8‐week period. High expression levels of EIF1AX were connected with dramatic tumour growth, and EIF1AX knock‐down significantly reduced tumour weight (Figure 2F,G).

### 3.3 Identification of p21 as a downstream target of EIF1AX

**Figure 3:**
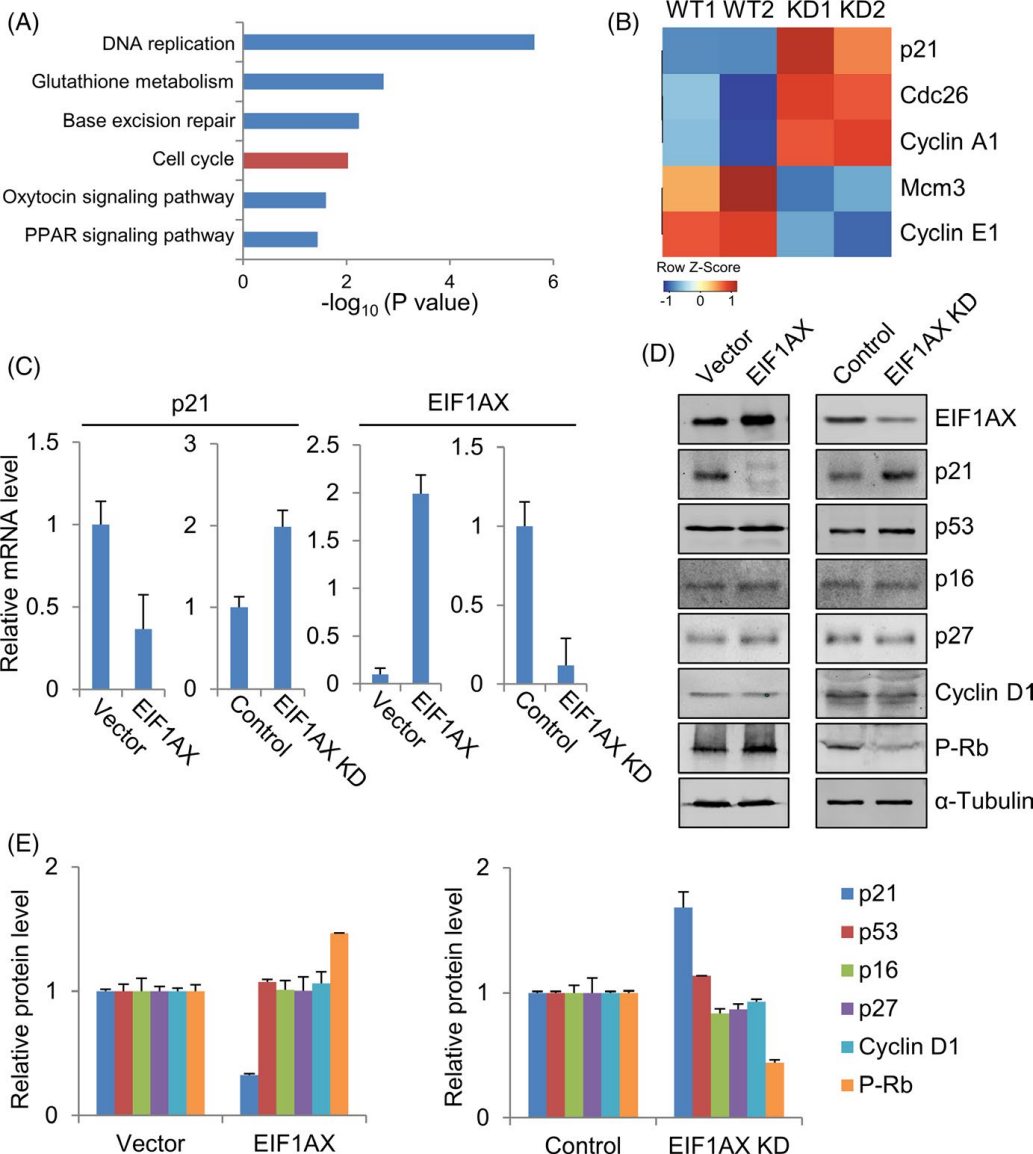
Identification of p21 as a downstream target of EIF1AX. A, B, EIF1AX knock‐down enhances *p21* gene expression, which inhibits cell cycle progression. Kyoto Encyclopedia of Genes and Genomes (KEGG) pathway (A) and Heat map analyses of abnormally expressed cell cycle‐related genes in EIF1AX knock‐down MCF‐7 cells (B) that were identified by the RNA‐Seq. C, qRT‐PCR analysis of p21 and EIF1AX in EIF1AX‐overexpressing or EIF1AX knock‐down cells. D, Western blotting analysis of the core proteins involved in G1/S transition in EIF1AX‐overexpressing or EIF1AX knock‐down cells. E, Quantification of proteins in (D)

To elucidate the underlying mechanisms by which EIF1AX promotes cell proliferation and breast tumorigenesis, we next performed RNA‐Seq analysis using the control and EIF1AX knock‐down MCF‐7 cells (Figure 3A,B). As expected, there were significant differences in gene expression profiles between the EIF1AX knock‐down and control MCF‐7 cells. Based on the gene expression dynamics, we found that the genes involved in the cell cycle showed abnormal expression in EIF1AX knock‐down MCF‐7 cells (Figure 3A,B).

To further confirm the EIF1AX‐mediated cell proliferation and tumorigenesis signaling pathways, we detected cell cycle‐associated genes expression by qRT‐PCR and Western blotting (Figure 3C‐E). As shown in Figure 3C‐E, although EIF1AX overexpression or knock‐down had no significant effect on the protein expression of several G1/S transition‐related genes, including p53, p16, p27 and cyclin D1, the RNA and protein expression of p21 were reduced in cells with EIF1AX overexpression and upregulated in cells with EIF1AX knock‐down. Consistent with the above results, the protein expression of p‐Rb, a downstream target of p21, was increased in cells with EIF1AX overexpression and reduced in cells with EIF1AX knock‐down. Our results demonstrate that the p21 gene is a downstream target of EIF1AX in breast cancer cells.

### 3.4 Transcriptional repression of p21 by EIF1AX

**Figure 4:**
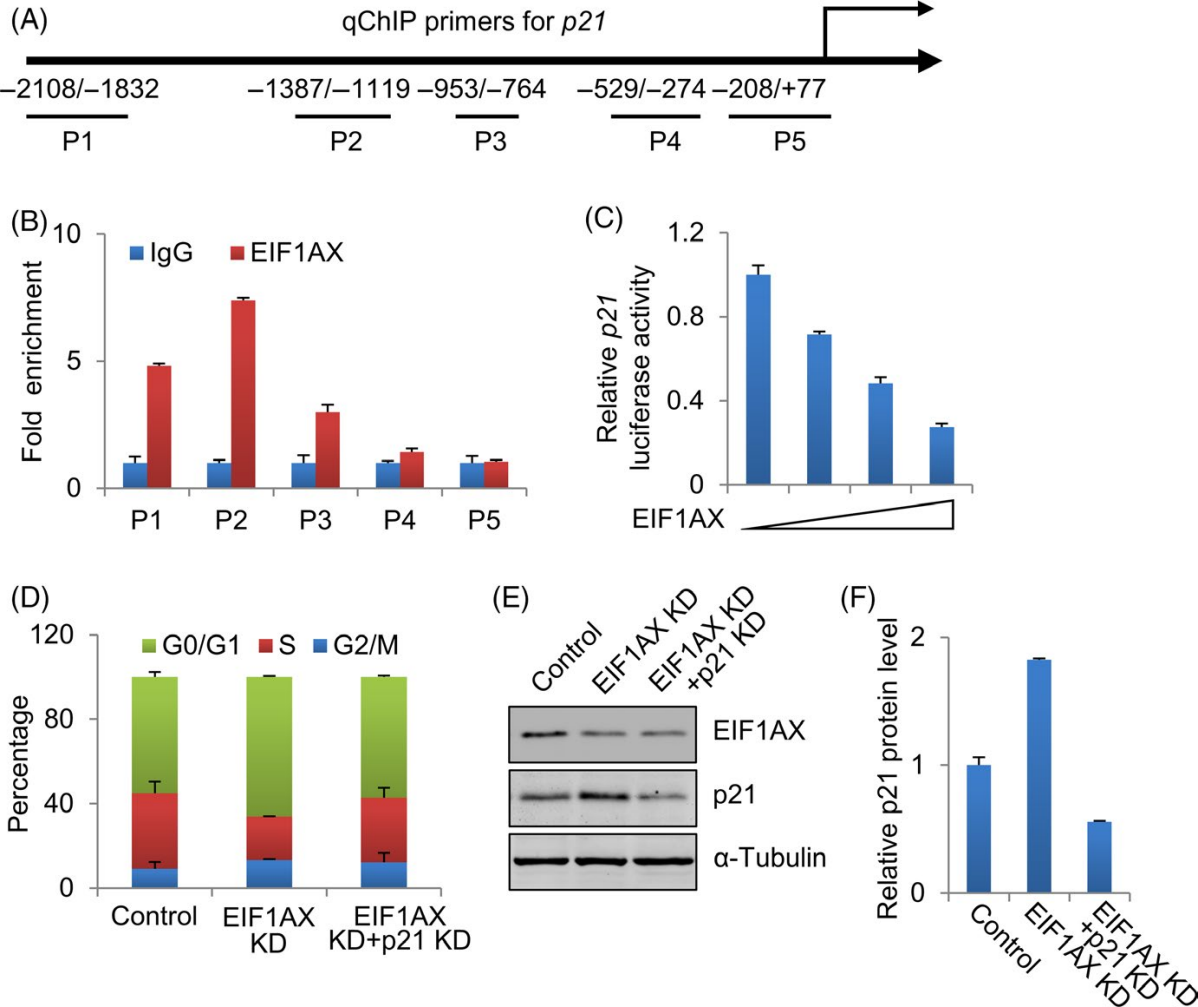
Transcriptional repression of p21 by EIF1AX. A, Diagram of human p21 promoter sequence. P1−5 show the regions of the p21 promoter detected by the paired primers. B, EIF1AX was recruited to p21 promoter. Soluble chromatin in MCF‐7 cells was immunoprecipitated with anti‐EIF1AX, rabbit normal IgG antibody served as control. qRT‐PCR analysis of the final extracted DNA using the primers specific to the proximal promoter region of the p21 gene. C, The effect of EIF1AX on p21 promoter activity. Luciferase assay of MCF‐7 cells transfected with p21‐Luc reporter construct and 0, 0.1, 0.2 and 0.4 µg/well EIF1AX expression construct. D, The effect of EIF1AX and p21 knock‐down on cell proliferation. Cell cycle analysis of control, EIF1AX knock‐down and EIF1AX‐ and p21‐knock‐down MCF‐7 cells by flow cytometry. The data represent the results from three independent experiments. E, Western blotting analysis of p21 in EIF1AX‐ and p21‐knock‐down MCF‐7 cells. F, Quantification of proteins in (E)

To determine whether EIF1AX regulates p21 gene transcription directly, ChIP assays were performed, and it was found that EIF1AX was recruited to the proximal region of the p21 promoter (Figure 4A,B). These results demonstrate that EIF1AX physically associates with the p21 promoter. To determine whether EIF1AX regulates the expression of p21 at the transcriptional level, we used luciferase assays to detect the effect of EIF1AX on the p21 promoter. As shown in Figure 4C, EIF1AX decreased the expression of the p21 gene promoter in a dose‐dependent way. These results indicate that EIF1AX downregulates p21 gene transcription.

We further determined the role of p21 downregulation in promoting EIF1AX‐dependent breast cancer cell proliferation by knocking down both p21 and EIF1AX to determine whether p21 knock‐down could rescue the EIF1AX knock‐down‐induced accumulation of cells in the G0/G1 phase. Either EIF1AX and p21 siRNA or EIF1AX siRNA and control siRNA as negative controls were co‐transfected into MCF‐7 cells. Flow cytometry analysis showed that p21 downregulation in EIF1AX‐downregulated cells resulted in a decreased percentage of cells in the G0/G1 phase (Figure 4D‐F). These results suggest that p21 is an important downstream mediator of EIF1AX in promoting G1/S transition and cell proliferation.

### 3.5 Transcriptional regulation of p21 by EIF1AX is p53‐independent

**Figure 5:**
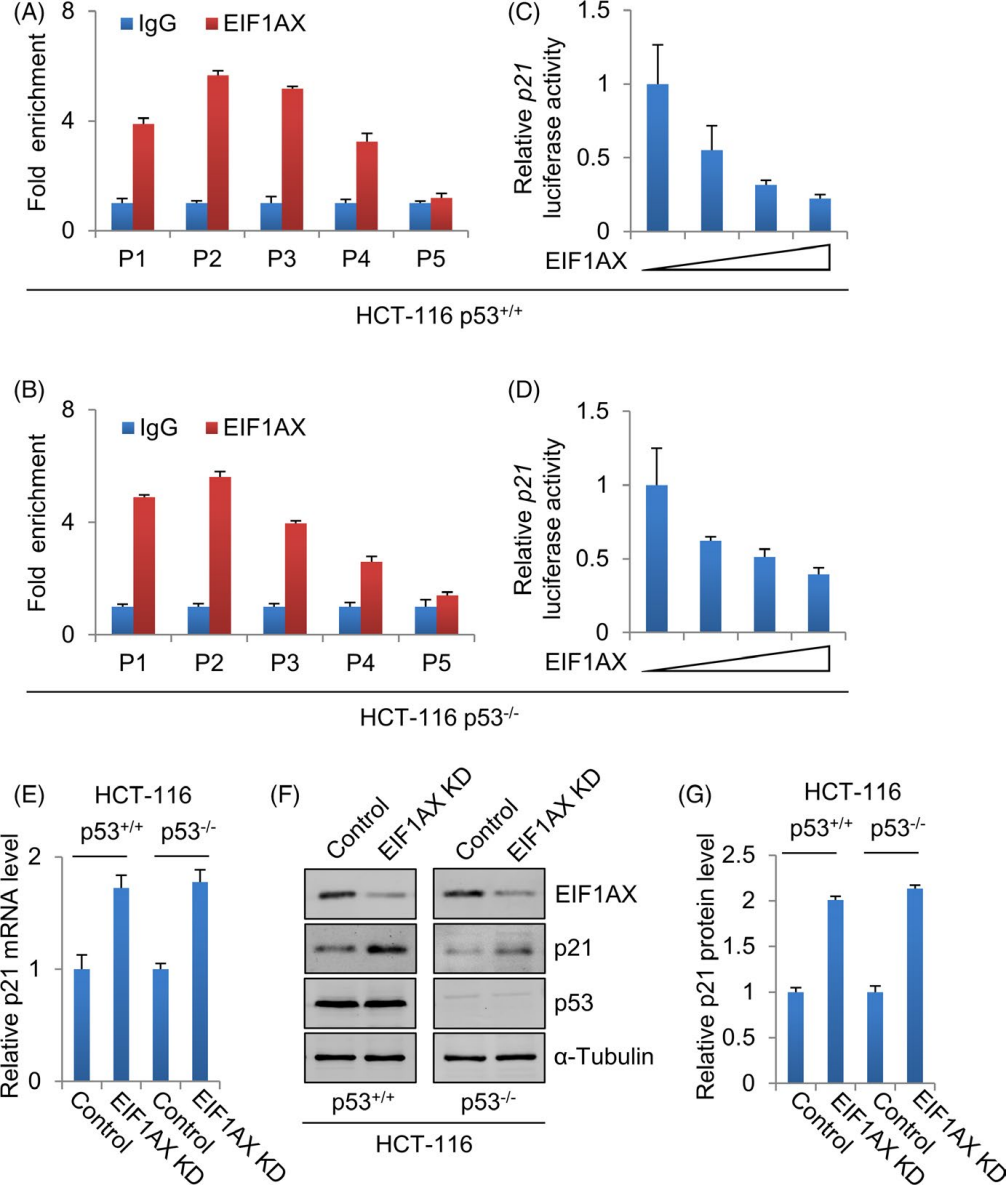
Transcriptional regulation of p21 by EIF1AX is p53‐independent. A, The recruitment of EIF1AX to the p21 promoter in p53^+/+^ HCT‐116 cells. Soluble chromatin from p53^+/+^ HCT‐116 cells was immunoprecipitated with anti‐EIF1AX, rabbit normal IgG antibody served as control. qRT‐PCR analysis of the final extracted DNA using the primers specific to the proximal promoter region of the p21 gene. B, The recruitment of EIF1AX to the p21 promoter in p53^−/−^ HCT‐116 cells. Soluble chromatin from p53^−/−^ HCT‐116 cells was immunoprecipitated with anti‐EIF1AX, rabbit normal IgG antibody served as control. The final extracted DNA samples were amplified by qRT‐PCR using primers specific to the proximal promoter region of the p21 gene. C, The effect of EIF1AX on p21 promoter activity in p53^+/+^ HCT‐116 cells. Luciferase assay of p53^+/+^ HCT‐116 cells transfected with p21‐Luc reporter construct and 0, 0.1, 0.2 and 0.4 µg/well EIF1AX expression construct. D, The effect of EIF1AX on p21 promoter expression in p53^−/−^ HCT‐116 cells. Luciferase assay of p53^−/−^ HCT‐116 cells transfected with p21‐Luc reporter construct and 0, 0.1, 0.2 and 0.4 µg/well EIF1AX expression construct. E, qRT‐PCR analysis of p21 in EIF1AX knock‐down p53^+/+^ (left panel) and p53^−/−^ (right panel) HCT‐116 cells. F, Western blotting analysis of p21 in EIF1AX knock‐down p53^+/+^ (left panel) and p53^−/−^ (right panel) HCT‐116 cells. G, Quantification of proteins in (F)

To confirm whether the transcriptional regulation of p21 by EIF1AX is p53‐dependent, ChIP assays were performed to confirm whether EIF1AX was directly recruited to the p21 promoter in p53^+/+^ HCT‐116 cells and p53^−/−^ HCT‐116 cells. EIF1AX was recruited to the proximal region of the p21 promoter in both p53^+/+^ and p53^−/−^ HCT‐116 cells (Figure 5A,B). These results demonstrate that EIF1AX, physically associated with the p21 promoter, functions in a p53‐independent manner. To determine whether EIF1AX regulates p21 transcriptional levels in a p53‐dependent manner, luciferase reporter assays were performed to examine the effect of EIF1AX on the p21 promoter in p53^+/+^ HCT‐116 cells and p53^−/−^ HCT‐116 cells. EIF1AX decreased the expression of p21 promoter in a dose‐dependent manner in both p53^+/+^ HCT‐116 cells and p53^−/−^ HCT‐116 cells (Figure 5C,D). Furthermore, the RNA and protein expression of p21 were increased in cells when EIF1AX was downregulated in both p53^+/+^ HCT‐116 cells and p53^−/−^ HCT‐116 cells (Figure 5E‐G). These results indicate that EIF1AX downregulates p21 gene transcription in a p53‐independent manner.

### 3.6 Clinicopathological significance of EIF1AX in breast cancer

**Figure 6:**
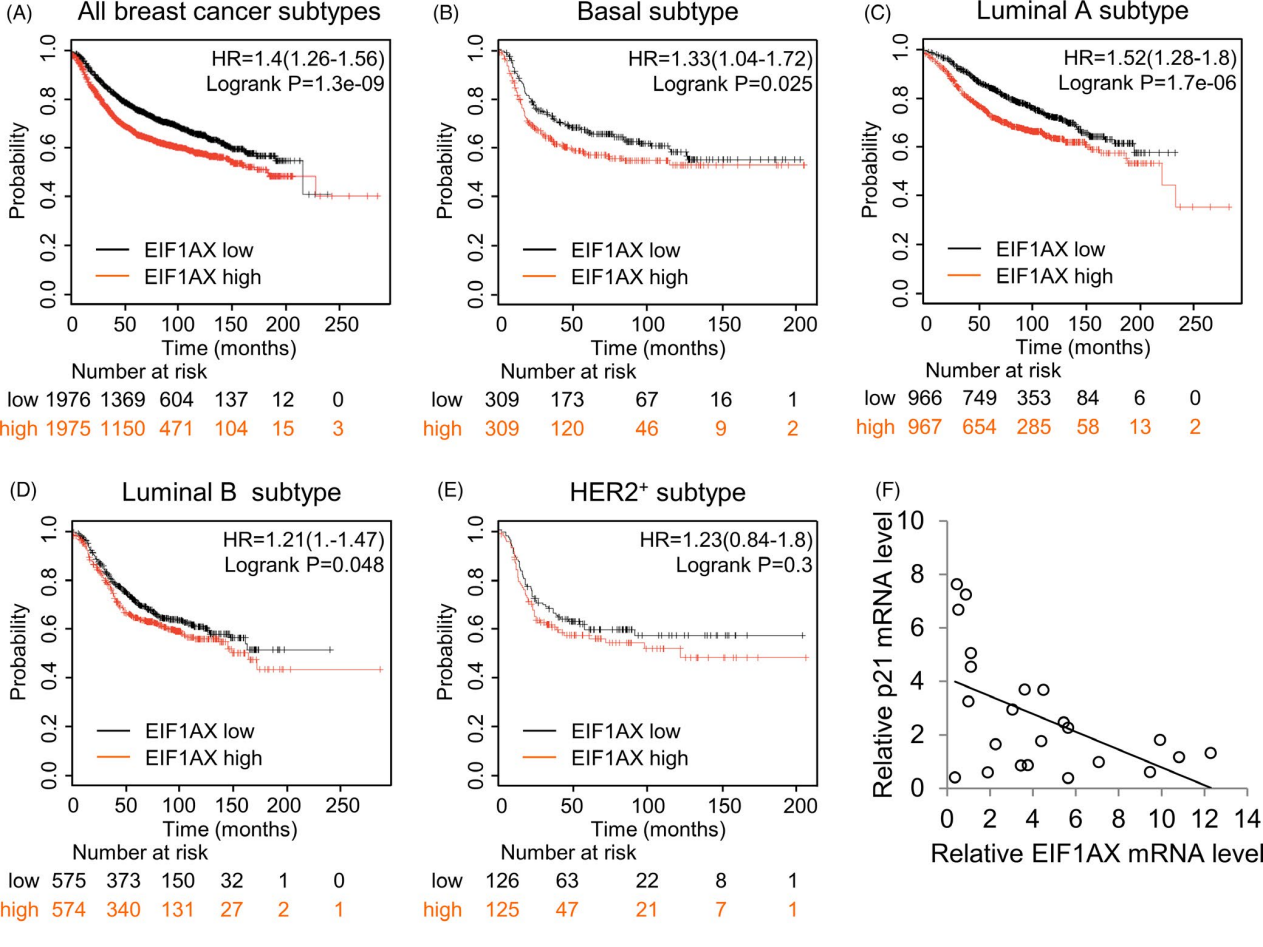
Clinicopathological significance of EIF1AX in breast cancer. A‐E, Analysis of relapse‐free survival of breast cancer patients using KM Plotter. The analysis included all breast cancer patients and patients with the basal, luminal A, luminal B or HER2^+^ subtypes. Hazard ratios and log‐rank *P* values are shown. F, Analysis of EIF1AX and p21 expression. Plots show the relative level of EIF1AX against that of p21

To inspect the clinical correlation of EIF1AX levels in the subtypes of human breast cancer, a comprehensive data set of 5143 breast cancer cases was analysed using the Kaplan‐Meier Plotter (KM Plotter) tool.^16^ These analyses indicate that elevated EIF1AX expression positively correlated with poor survival in patients with breast carcinoma (Figure 6A). There were strongest associations among the basal, luminal A and luminal B breast cancer subtypes (Figure 6B‐D). A similar effect was observed for the HER2^+^ subtype, although with a lower significance, owing to the substantially smaller sample size in this setting (Figure 6E). Furthermore, we found that p21 mRNA levels in tumour tissues were negatively related to EIF1AX levels (*P* < .01), which demonstrates that EIF1AX was negatively correlated with p21 expression levels in breast cancer (Figure 6F). Together, these findings point towards a tumour‐promoting role of EIF1AX in breast cancer.

## 4 DISCUSSION

EIF1AX is the only PIC subunit known to be recurrently mutated in cancer to date. EIF1AX mutations were initially discovered in uveal melanomas^7^ and reported in 11% of poorly differentiated thyroid cancers (PDTCs) and anaplastic thyroid cancers (ATCs), and almost invariably associated with oncogenic RAS mutations.^11^, ^17^ These mutations synergistically drive the occurrence of thyroid tumours through the ATF4 and c‐MYC pathways.^18^ These results imply that EIF1AX may have an important effect on tumour progression. However, the expression and function of EIF1AX in breast cancer have not yet been investigated. In this study, we show that levels of EIF1AX are significantly upregulated in breast cancer samples and that there is a positive correlation between EIF1AX expression levels and worse survival rates for patients with breast cancer.

Playing key roles in cell migration, cell cycle regulation and apoptosis,^19^ p21 is intricately regulated in a p53‐dependent or independent manner.^20^ Herein, by using a cancer cell model with p53 deficiency, we show that EIF1AX induces the transcriptional repression of p21 in a p53‐independent manner. It is known that a high prevalence of p53 mutations exists in almost all types of cancers. Additionally, our data show that EIF1AX is highly expressed in breast cancer cell lines with the p53 mutation (BT‐474, T47D and MDA‐MB‐231 cells; Figure 1A,B). Hence, our findings suggest that EIF1AX probably retains its cancer cell growth‐promotion capacity even in p53‐mutated cancer cells.

Translation initiation is orchestrated through the precise regulation of cap binding and the 43S PIC. Deregulation of translation initiation is common in tumorigenesis. In many cancers, the expression of the components of the EIF4F cap‐binding complex is increased, such as EIF4E, EIF4A and EIF4G.^21^, ^22^, ^23^, ^24^ C‐MYC controls the transcriptional levels of these genes.^25^, ^26^ Overexpression of EIF4E in cultured cells can induce transformation^27^ and accelerate tumorigenesis in genetically engineered mouse models.^28^, ^29^ EIF4E is frequently found to be overexpressed in cancer cells and may help increase a translational output that supports tumorigenesis.^30^ EIF1AX is a translation initiation component; here, we show that EIF1AX is involved in the transcriptional regulation of p21. Our study identifies the transcription control facet of the translation initiation component in tumorigenesis. In this study, we found that EIF1AX promoted the occurrence of breast cancer through transcriptional regulation of cell cycle elements. However, as a translation initiation factor, EIF1AX may also promote the occurrence of breast cancer by influencing the translation of other key factors, which can be further explored in future studies.

In summary, we show that EIF1AX expression is elevated in breast carcinomas and there is positive correlation between EIF1AX expression level and poor survival rates of breast cancer patients. We demonstrate that EIF1AX facilitates cell proliferation of breast cancer and tumorigenesis, possibly by promoting the G1/S transition. We identified p21 as a downstream target of EIF1AX in breast cancer cells. These results imply that EIF1AX may have an important effect on the development and promotion of breast cancer; thus, EIF1AX may serve as a valuable target for breast cancer therapy.

## CONFLICT OF INTEREST

The authors declare no conflicts of interest.

## AUTHOR CONTRIBUTIONS

YZ conceived and designed the experiments. YZ, YL, LG, SY and GF performed the research and analysed the data. YZ and GF interpreted the data. YZ wrote the paper. All authors have read and approved the final manuscript.

## Supporting information

Table S1

## Data Availability

The data that support the findings of this study are available from the corresponding author upon reasonable request.
